# *Bacteroidales* species in the human gut are a reservoir of antibiotic resistance genes regulated by invertible promoters

**DOI:** 10.1038/s41522-021-00260-1

**Published:** 2022-01-10

**Authors:** Wei Yan, A. Brantley Hall, Xiaofang Jiang

**Affiliations:** 1 National Library of Medicine, National Institutes of Health, Bethesda, Maryland USA; 2 Department of Cell Biology and Molecular Genetics, University of Maryland, College Park, Maryland USA; 3 Center for Bioinformatics and Computational Biology, University of Maryland, College Park, Maryland USA

**Keywords:** Molecular evolution, Microbial genetics, Bacteria

## Abstract

Antibiotic-resistance genes (ARGs) regulated by invertible promoters can mitigate the fitness cost of maintaining ARGs in the absence of antibiotics and could potentially prolong the persistence of ARGs in bacterial populations. However, the origin, prevalence, and distribution of these ARGs regulated by invertible promoters remains poorly understood. Here, we sought to assess the threat posed by ARGs regulated by invertible promoters by systematically searching for ARGs regulated by invertible promoters in the human gut microbiome and examining their origin, prevalence, and distribution. Through metagenomic assembly of 2227 human gut metagenomes and genomic analysis of the Unified Human Gastrointestinal Genome (UHGG) collection, we identified ARGs regulated by invertible promoters and categorized them into three classes based on the invertase-regulating phase variation. In the human gut microbiome, ARGs regulated by invertible promoters are exclusively found in *Bacteroidales* species. Through genomic analysis, we observed that ARGs regulated by invertible promoters have convergently originated from ARG insertions into glycan-synthesis loci that were regulated by invertible promoters at least three times. Moreover, all three classes of invertible promoters regulating ARGs are located within integrative conjugative elements (ICEs). Therefore, horizontal transfer via ICEs could explain the wide taxonomic distribution of ARGs regulated by invertible promoters. Overall, these findings reveal that glycan-synthesis loci regulated by invertible promoters in *Bacteroidales* species are an important hotspot for the emergence of clinically-relevant ARGs regulated by invertible promoters.

## Introduction

The proliferation of ARGs has compromised antibiotic treatment for bacteria infections^1^. The human gut microbiome is an important reservoir of ARGs^2–4^ and the spread of ARGs from gut microbes to pathogens has been documented^5^. Therefore, ARGs in the human gut microbiome pose a growing threat to human health.

Often, bacteria-carrying ARGs are outcompeted by susceptible strains due to the costs associated with the maintenance and expression of the ARGs^6–8^. Though it is costly, bacteria can ameliorate the fitness costs of maintaining ARGs through different strategies^9^, such as no-cost, low-cost or gain-of-fitness mutations^10,11^, compensatory mutations at a second site^12–14^, or genetic coselection of resistance genes in genetic linkage^15,16^. Phase-variable antibiotic resistance, which was only recently reported^17^, is a newly identified mechanism for antibiotic resistant bacteria to mitigate the fitness cost of encoding ARGs.

Phase variation refers to a reversible change that generates phenotypic variation that helps bacteria adapt to rapidly changing environments^18,19^. Phase variation often manifests through reversible inversion of DNA regions containing promoters such that in one orientation, a downstream gene is expressed, while in the alternate orientation, the downstream gene is not expressed^17^. Such DNA inversions are generally mediated by invertases, which recognize inverted repeats flanking the invertible region and catalyze the reversible inversion^20–22^. Genes regulated by invertible promoters often contribute to the regulation of characteristics important for bacterial colonization and virulence, including fimbriae^23,24^, flagella^25^, and capsular polysaccharides^26,27^.

Recent advances in computational methods have contributed to the effective identification of the intergenic invertible DNA regions in microbial genomes^17,28^. ARGs were found to be regulated by invertible promoters in certain human gut bacteria^17^. Bacteria with ARGs regulated by invertible promoters in the ON orientation could be selected for in the presence of antibiotics. Bacteria with ARGs regulated by invertible promoters in the OFF orientation could ameliorate the fitness cost in the absence of antibiotics, which facilitate the maintenance of these ARGs for longer periods in microbial communities^17^. The emergence of ARGs regulated by invertible promoters likely increases the burden of combating antibiotic resistance and the spread of these ARGs to pathogens poses an increasing threat to human health. However, many questions that are fundamental to assess the risk of ARGs regulated by invertible promoters remain unanswered, specifically (1) what is the diversity of the invertible promoters regulating ARGs (IP-ARG)? (2) what are the taxonomic boundaries and geographic distribution of the IP-ARG? and (3) how did the IP-ARG originate?

Taking advantage of the considerable amount of human gut metagenomic data generated during the last decade, we aimed to address these questions. Here, we systematically searched for ARGs regulated by invertible promoters through metagenomic assembly of 2227 human gut metagenomes and the Unified Human Gastrointestinal Genome (UHGG) collection of human gut genomes. We found that the taxonomic distribution of ARGs regulated by invertible promoters appears to be restricted to the order *Bacteroidales*. Genomic analysis showed that ARGs regulated by invertible promoters have been convergently derived from ARG insertions into glycan-synthesis loci regulated by invertible promoters. Notably, the identified ARGs regulated by invertible promoters were found to have been mobilized through ICEs and have been widely geographically distributed. Our results reveal the prominent role of glycan-synthesis loci regulated by invertible promoters in *Bacteroidales* species in providing hotspots for the emergence of clinically relevant ARGs regulated by invertible promoters.

## Results

### Identification and classification of invertible promoters regulating ARGs

To expand the known repertoire of ARGs regulated by invertible promoters, we searched publicaly available human gut metagenomic datasets, comprising a total of 2227 samples, using the tool PhaseFinder (Supplementary Data 1). Briefly, we assembled the metagenomic data and then searched for regions regulated by invertible promoters with PhaseFinder. PhaseFinder first detects putative invertible regions in the assemblies by identifying inverted repeats, mimics their inversion *in silico*, then checks if there are metagenomic reads supporting both potential orientations to determine whether inversion occurs. We then identified putative invertible promoters in the regions by searching for promoter sequences in the identified invertible regions. We then scanned the operons downstream of the invertible promoters to find those containing ARGs. This search uncovered 62 contigs, from samples collected from 47 individuals, containing invertible promoters regulating ARGs (IP-ARG). Redundant instances in the same individual were removed. In total, 48 IP-ARG instances were identified. All identified IP-ARG were located immediately downstream of a gene encoding an invertase and have promoters with an identical or close match to the consensus-promoter sequence -33 (TTTG) and -7 (TANNTTTGY)^29^.

**Fig. 1:**
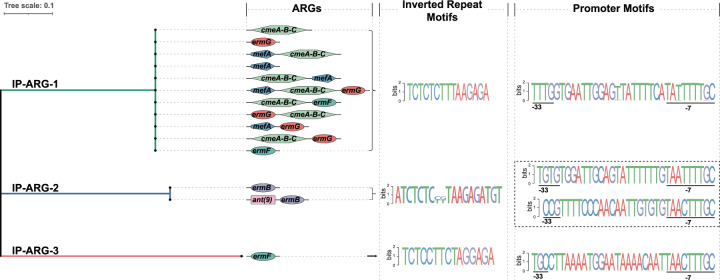
Three classes of invertible promoters regulating ARGs (IP-ARG) (IP-ARG-1, IP-ARG-2, and IP-ARG-3). The ARGs and putative ARG organization patterns are shown for each class. Different ARGs are shown in different shapes and colors.

We constructed a phylogenetic tree based on the nucleotide sequence of the invertases. We found that the invertible promoters regulating ARGs can be grouped based on the invertase into three distinct classes, denoted IP-ARG-1, IP-ARG-2, and IP-ARG-3 (Fig. 1, Supplementary Data 2). The nucleotide sequences of the invertase of the same class are nearly identical. Of the three classes, class IP-ARG-1 was found to contain the most identified instances (42 out of 48).

To further characterize these three classes of IP-ARG, we analyzed the motifs of inverted repeats and invertible promoters as well as the ARGs regulated by the invertible regions. The invertible promoter motifs varied among classes but were nearly identical within the same class (Fig. 1). The inverted repeat motifs were found to be similar across different classes. Interestingly, we found two invertible regions located immediately downstream of the invertase gene in class IP-ARG-2 (Fig. 1). The two invertible regions were located adjacent to each other and the inverted repeats were similar in these two regions. This suggested that these two invertible regions could be regulated by the same upstream invertase gene. However, the invertible promoters were different from each other (Fig. 1, Supplementary Data 2). This suggested that there may be differences in the strength of the promoters leading to differences in expression level. The ARGs regulated by invertible promoters varied among classes (Fig. 1, Supplementary Data 2). Most invertible promoters of class IP-ARG-1 regulated the *cmeABC* operon, *ermG* gene, or both, while some also regulated other ARGs, including *mefA*, *ermF*, or *tetQ*. These ARGs could confer resistance to diverse antibiotics, such as fluoroquinolone, macrolides, lincosamides, streptogramin, and tetracycline^30–34^. The *ermB* and *ermF* genes, which confer resistance to streptogramin, macrolides, and lincosamides^31,33^, were found to be regulated by class IP-ARG-2 and class IP-ARG-3, respectively. In addition, class IP-ARG-2 was also found to regulate an *ant(9)* gene homolog which confers resistance to aminoglycosides^35,36^.

We analyzed the promoter orientation in the metagenomic samples and found that in only 4 out of 96 cases (with total supporting reads >20), the promoters were predominantly in the ON orientation while the remainder were in the OFF orientation (Supplementary Data 3). Of the four cases, three have been reported previously^17^ and promoters in the ON orientation have been found to be selected for by antibiotics, while the fourth has no metadata available in terms of antibiotic treatment. This supports that in all the four types of invertible promoters, the OFF orientation is advantageous in most samples, possibly by mitigating the fitness cost of expressing ARGs in the absence of antibiotics.

### Taxonomic distribution of IP-ARG

We expanded the search to include genomes from the Unified Human Gastrointestinal Genome (UHGG) collection^37^ by detecting nearly identical (>99% identity) invertase genes that are immediately upstream of invertible promoters regulating ARGs. These invertase genes were grouped into the corresponding classes of IP-ARG (Supplementary Data 4). Consistent with the finding from metagenomic contigs, most ARGs regulated by invertible promoters belonged to class IP-ARG-1 (176 out of 210 genomes). Based on the metadata from the metagenomic samples used in this study and provided by the UHGG collection, we found that ARGs regulated by invertible promoters were observed in multiple countries, but the geographic prevalence varied across classes (Table 1, Supplementary Data 2 and 4). Examples of IP-ARG-1 and IP-ARG-2 were identified in metagenomes from 17 and 7 countries, respectively, spanning three continents (Asia, Europe, and North America). Class IP-ARG-3 were only observed in the metagenomes from Denmark, which might be due to the limited sampling of publically-available metagenomic data. The results reveal that species harboring ARGs regulated by invertible promoters are widely geographically distributed.

**Table 1:** Taxonomic and geographic distribution of ARGs regulated by invertible promoters.

IP-ARG group^a^	Order^b^	Family	Genus	Species	Country
1	*Bacteroidales*	*Bacteroidaceae*	*Bacteroides*	*B. cellulosilyticus*, *B. eggerthii*,	Austria, Canada, China, Denmark, Estonia, Finland, France, Germany, Israel, Japan, Kazakhstan, Netherlands, Russia, Spain, Sweden, United Kingdom, United States
				*B. fluxus*, *B. fragilis*,	
				*B. intestinalis*, *B. ovatus*,	
				*B. stercoris*, *B. thetaiotaomicron*,	
				*B. uniformis*	
			*Phocaeicola*	*P. coprocola*, *P. coprophilus*,	
				*P. dorei*, *P*. sp000432735,	
				*P. vulgatus*	
			*Prevotella*	*P. copri*, *P*. sp000834015,	
				*P*. sp001275135,	
				*P*. sp900313215, *P. stercorea*	
		*Barnesiellaceae*	*Barnesiella*	*B. intestinihominis*	
		*Coprobacteraceae*	*Coprobacter*	*C. fastidiosus*	
		*Tannerellaceae*	*Parabacteroides*	*P. distasonis*, *P. goldsteinii*, *P. merdae*	
2	*Bacteroidales*	*Bacteroidaceae*	*Bacteroides*	*B. caccae*, *B. eggerthii*,	China, Denmark, Estonia, Israel, Italy, United Kingdom, United States
				*B. fragilis*,	
				*B. thetaiotaomicron*,	
				*B. uniformis*	
			*Paraprevotella*	*P. xylaniphila*	
			*Phocaeicola*	*P. dorei*	
3	*Bacteroidales*	*Bacteroidaceae*	*Phocaeicola*	*P. dorei*	Denmark
		UBA932	RC9	RC9 sp000434935	

^a^The class of invertible promoters regulating ARGs (IP-ARG).

^b^Taxonomic information of the bacteria host at order, family, genus, and species levels. The taxonomic information is annotated based on GTDB release95.

IP-ARG was found to be exclusively distributed *Bacteroidales* species (Table 1, Supplementary Data 2 and 4). Class IP-ARG-1 was observed in 24 *Bacteroidales* species from the families *Bacteroidaceae*, *Barnesiellaceae*, *Coprobacteraceae*, and *Tannerellaceae*. Class IP-ARG-2 could be identified in 7 species from *Bacteroidaceae* that belonged to the order *Bacteroidales*. Class IP-ARG-3 was found in two species, *Phocaeicola dorei* and a novel species RC9 sp000434935, which also belongs to the *Bacteroidales* order. The wide taxonomic distribution combined with the sparse occurrence of these ARGs and the presence of nearly identical invertases in a broad range of *Bacteroidales* hosts is not consistent with vertical transmission, suggesting that ARGs regulated by invertible promoters could be horizontally transferred by mobile genetic elements (MGEs).

### All three classes of IP-ARG are located within integrative conjugative elements

**Fig. 2:**
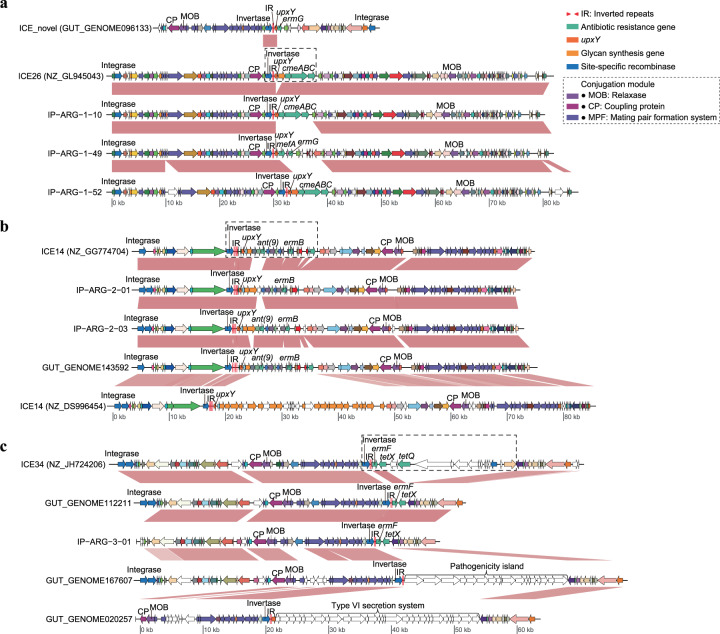
Genomic comparison and context analysis of representative contigs carrying IP-ARG-1 (a), IP-ARG-2 (b), and IP-ARG-3 (c) as well as related elements. The regions located adjacent to the invertible promoters (black-dotted boxes) were found to be highly variable across element variants within each ICE. ICE26, ICE14, and ICE34 are ICE accession numbers from ImmeDB. ICE_novel1 is a newly identified ICE that has not been included in the ImmeDB database. The NCBI genome-accession numbers are shown in the parentheses after the ICE accession numbers. The sequence labels that start with GUT_GENOME are genome-accession numbers of the UHGG database. Orthologous genes are plotted with the same color and are linked by pink connections. Site-specific recombinase, antibiotic resistance, and glycan-synthesis genes are colored blue, light green, and orange, respectively. The genes that do not have orthologs are white.

To determine whether IP-ARG was on MGEs, we searched the invertases and invertible regions regulating ARGs as well as the flanking sequences against ImmeDB^38^ and the ICEberg database^39^. We found that all three classes of IP-ARG are located within integrative conjugative elements (ICEs) (Fig. 2, Supplementary Fig. 1, and Supplementary Data 4). The majority of examples from class IP-ARG-1 were identified within ICEs related to ICE26 in ImmeDB (Fig. 2a). ARGs regulated by invertible promoters from class IP-ARG-1 were also found within another novel ICE (Fig. 2a). This suggests that the progenitor of IP-ARG-1 might have emerged before subsequently inserting into multiple ICEs. Examples of class IP-ARG-2 were detected in ICEs related to ICE14 in ImmeDB (Fig. 2b). Class IP-ARG-3 that is contained within an ~50-kb sequence fragment most closely matches ICE34 in ImmeDB (Fig. 2c). The fact that all three classes of IP-ARG are located within ICEs may allow for further dissemination in the human population because ICEs encode the necessary machinery to horizontally transfer between species mobilizing any ARGs that they acquire.

ARGs regulated by invertible promoters were frequently observed to be included in a highly variable region that was located immediately downstream of the invertible regions. The highly varied regions contained genes that were not necessary for the ICE replication and transfer, but often important for conferring selective and adaptive advantages for hosts in the changing environments (Fig. 2). Most of the variable genes in the highly varied region were ARGs in ICEs containing class IP-ARG-1 (Fig. 2a), which suggested multiple insertions of ARGs at this region. In ICEs carrying class IP-ARG-2, not only the ARGs but also the glycan-synthesis genes, even clusters that contained only glycan-synthesis genes, were located in the regions downstream of the invertases (Fig. 2b). In different ICEs with class IP-ARG-3, the highly variable regions contained varied genes or operons, such as ARGs, operons involved in pathogenicity island or T6SS, or integrative and mobilizable elements encoding ARG *tetQ* (Fig. 2c). All these indicated that the loci in such highly variable regions downstream of the invertible promoters were hotspots for the acquisition of elements, including ARGs. The newly acquired ARGs can then be subsequently horizontally transferred to a wide range of *Bacteroidales* species, serving as a reservoir.

### The invertible regions regulating ARGs appear to originate from those regulating glycan-synthesis genes independently and convergently

**Fig. 3:**
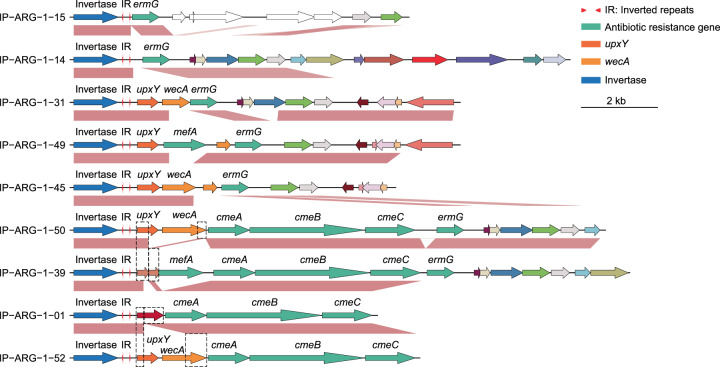
Comparisons of highly variable regions carrying IP-ARG-1 demonstrate the degeneration of the *upxY* gene. Different degeneration statuses of the *upxY* gene are: completely degenerated (IP-ARG-1–14 and IP-ARG-1–15), partially degenerated (IP-ARG-1–39), fusion gene (IP-ARG-1–01), and intact gene (the remainder). Orthologous genes are plotted with the same color and are linked by pink connections. Site-specific recombinase, antibiotic resistance, and glycan-synthesis genes are colored blue, light green, and orange, respectively. The genes that do not have orthologs are white.

To better understand the evolution of IP-ARG, we performed comparative analyses on the highly varied regions regulated by invertible promoters. We found that glycan-synthesis genes, such as *wecA* gene, or genes involved in synthesis or regulation of capsular polysaccharide synthesis, such as the *upxY* gene^40^, were frequently located immediately downstream of the invertible promoters and upstream of the identified ARGs (Figs. 2a, b and 3). In addition, the inverted repeats of class IP-ARG-2 in the genome GUT_GENOME143592 are identical to the inverted repeats of the invertible promoter regulating glycan synthesis cluster on ICE14 (NZ_DS996454). The invertase amino acid sequences of these two are more than 96% identical based on BLAST analysis^41^. These results support that the invertible regions regulating ARGs share a common evolutionary origin with invertible regions regulating glycan-synthesis genes.

Gene-context analysis further shows that invertible regions regulating ARGs appeared to be derived from invertible regions originally regulating glycan-synthesis genes. ARGs regulated by invertible promoters were frequently found to be located mostly downstream or in a few cases upstream of glycan-synthesis genes (Figs. 2a, b and 3). The *upxY* gene, a gene that is often the first gene in polysaccharide-biosynthesis operons, is also the first gene in the several ARG operons regulated by invertible promoters (Fig. 3). BLASTn comparisons between the highly variable regions carrying IP-ARG-1, showed that it is likely that *upxY* is completely degenerated in IP-ARG-1–14 and IP-ARG-1–15, partially degenerated in IP-ARG-1–39, and fused with the partial sequence of the *wecA* gene in IP-ARG-1–01 (Fig. 3).

**Fig. 4:**
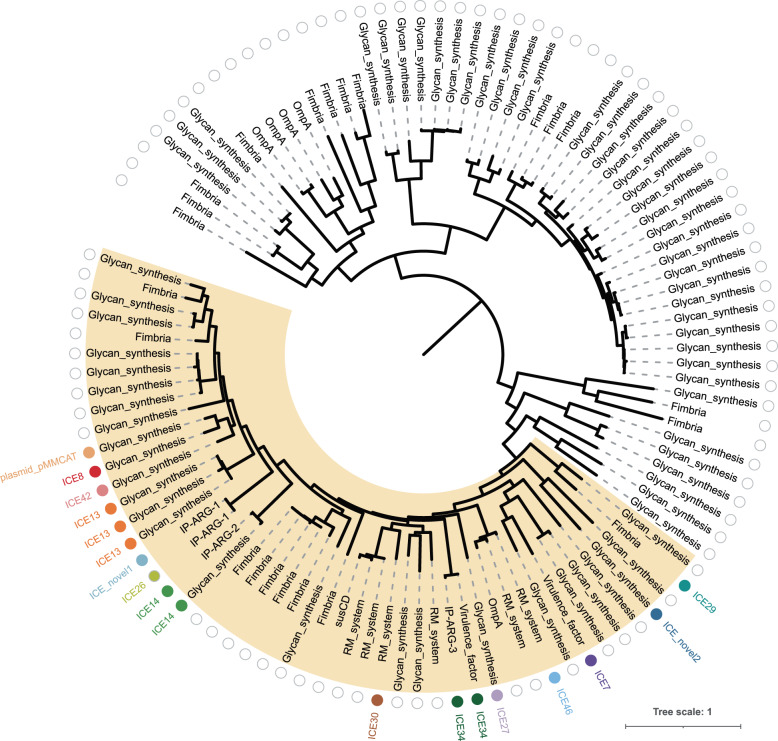
The evolutionary events that led to the emergence of ARGs regulated by invertible promoters may have occurred independently at least three times as the result of convergent evolution. The phylogenetic tree is inferred based on the alignment of protein sequences of invertases. The invertase tree is rooted with *Bacteroidales* tyrosine integrases as the outgroup. Invertases are labeled with the functional annotation of the loci regulated by the invertase and invertible promoters. The empty gray circle indicates that the invertase is not on a conjugative element, while the solid-colored circle indicates that the invertase is on a conjugative element. Different conjugative elements are distinguished by different colors. The plasmid in the tree is a conjugative plasmid pMMCAT^69^. The ICE names are based on the ImmeDB database and the ICE_novel1 and 2 are newly identified ICEs that have not been included in the ImmeDB database. The clade where invertases were frequently mobilized by conjugative elements is colored light yellow.

To understand the evolutionary history of different classes of IP-ARG, a phylogenetic tree was constructed based on the protein sequences of the local invertase genes (Fig. 4). We rooted the invertase phylogenetic tree with *Bacteroidales* tyrosine integrases as the outgroup and found that the invertases of all three classes of IP-ARG are located in a clade where invertases were frequently mobilized by conjugative elements. Invertases of class IP-ARG-1 were found in two different ICEs, likely indicating that the ARG was inserted adjacent to the invertase before they were transferred together to another ICE. The closest relative of class IP-ARG-2 is an invertase of glycan-synthesis locus, which is also located on the ICE of the ICE14 family. Given the fact that neighboring branches are mostly glycan-synthesis loci, the most parsimonious explanation is that class IP-ARG-2 emerged as the ARG inserted into the glycan-synthesis operon localized in ICEs. Class IP-ARG-3 branches early in the tree, suggesting that the emergence of class IP-ARG-3 was likely independent of class IP-ARG-1 or IP-ARG-2. Hence, the emergence of different classes of IP-ARG might be the result of convergent evolution and the evolutionary events that led to the emergence of ARGs regulated by invertible promoters likely occurred independently at least three times. Given the large variety of glycan-synthesis loci regulated by invertible promoters in *Bacteroidales* species, this finding suggests that new classes of IP-ARG might readily emerge.

## Discussion

Our study is a comprehensive investigation of the origin, evolution, and prevalence of invertible promoters regulating ARGs. Our analysis revealed that ARGs regulated by invertible promoters are (a) exclusively found in *Bacteroidales* species, (b) often originate from ARG insertions into glycan-synthesis loci regulated by invertible promoters, (c) frequently mobilized by ICEs that may explain their wide taxonomic distribution within the *Bacteroidales* and their rapid dissemination, and (d) widely geographically distributed.

The IP-ARG found in this study was based on in silico prediction and further experiments are needed to confirm if these ARGs are indeed subject to phase variation. In the previous study, the phase-variable expression of ARGs regulated by invertible promoters was experimentally verified^17^. In this study, several of the identified ARGs are farther from the promoter, and in some cases, the *upxY* gene downstream of the invertible promoter region is degenerated. Due to its role in transcriptional antitermination, degeneration of *upxY* may lead to ARGs regulated by invertible promoters not being expressed^40^. Our analysis only identified ARGs regulated by invertible promoters in *Bacteroidales* species. There could be several reasons for this observed taxonomic restriction. First, gut *Bacteroidales* genomes typically contain numerous loci regulated by invertible promoters. In some *Bacteroides* species, such as *Bacteroides fragilis*, there are more than 20 such loci including up to 7 capsular polysaccharide loci^42,43^. While other taxa prevalent in the gut, including Proteobacteria, Firmicutes, and Actinobacteria, have loci regulated by invertible promoters, these loci are far less common and have far fewer examples per genome^17,44^. Therefore, ARG insertions into *Bacteroidales* loci regulated by invertible promoters are more probable than insertions into loci regulated by invertible promoters in other phyla. Furthermore, as abundant members of the human gut microbiome, metagenomic sequencing leads to high coverage of *Bacteroidales* species, which increases the likelihood that a region regulated by invertible promoters can be detected with PhaseFinder^17^. Though more than two thousand metagenomic samples were analyzed, no ARGs regulated by invertible promoters were identified in other taxa. This might be due to the limitation of PhaseFinder or suggests that ARGs regulated by invertible promoters might be less common in taxa other than *Bacteroidales*.

This finding highlights a potential threat to human health that *Bacteroidales* species pose as a reservoir for the dissemination of ARGs regulated by invertible promoters. *Bacteroidales* is one of the most abundant taxa in the human gut and *Bacteroidales* species are regarded as a reservoir of ARGs^45^. Moreover, members of *Bacteroidales* species, such as *Bacteroides fragilis*, are considered opportunistic pathogens^46^ and can be the causative agent of appendicitis and intra-abdominal abscesses^47,48^. Antibiotics have been used to treat such infections, but an increasing rate of antibiotic resistance has been noted in *Bacteroidales* species^49–52^. ARGs regulated by invertible promoters may contribute to the continued maintenance of clinically relevant ARGs such as *cmeABC*, *ermF*, and *tetQ*, which confer resistance to widely used antibiotics including macrolides, streptogramin and tetracycline^30,33,34^. Most species identified with ARGs regulated by invertible promoters are considered symbionts in the human gut. However, ARG transfer from symbionts to pathogens through horizontal transfer has been documented^5,53,54^. The transfer of ARGs regulated by invertible promoters from symbionts to pathogens via MGEs, such as the ICEs identified in this study, might promote resistance to a wide array of antibiotics in pathogenic species^55^, posing a threat to public health in the future. Due to the fact that the transfer of ICEs from *Bacteroidales* to other orders is rare^38^, the spread of ARGs regulated by invertible promoters is likely to be limited within the order *Bacteroidales*. As such, monitoring these *Bacteroidales* species, especially the ICEs in these species, might be important in mitigating the threat of ARGs regulated by invertible promoters to human health.

## Methods

### De novo assembly and gene annotation of metagenomic datasets

We downloaded metagenomic sequencing data that consisted of 2227 human gut samples encompassing seven studies (Supplementary Data 1). Low-quality reads were removed and sequencing adapters were trimmed with trim_galore (v0.6.4, https://github.com/FelixKrueger/TrimGalore). The filtered data were mapped to the human genome (hg19) using bowtie2 (v2.3.5.1)^56^ to filter human reads. The cleaned reads from each sample were assembled with SPAdes (v3.14.0) using the --meta option^57,58^, and sequences less than 500 bp were removed. Gene annotation was performed using Prokka (v1.14.5)^59^ with the parameter --metagenome.

### Identification of putative invertible promoters

PhaseFinder (v1.0)^17^ was employed to identify putative invertible regions in the metagenomic assemblies. The default parameters of PhaseFinder were used. We filtered the results by removing the invertible DNA regions with <2 reads supporting the R orientation from the paired-end method, and the Pe_ratio <1%. Furthermore, the invertible DNA regions containing or overlapping coding sequences (CDS) were removed. We used FIMO^60^ to search for promoter sequences in invertible regions based on previously identified promoter motifs^17^. Only invertible regions with promoters detected were retained for further analysis. The sequence-motif logos of aligned promoters and aligned inverted repeats were generated with WebLogo (version 2.8.2)^61^.

### Annotation of ARGs

We extracted the genes in the operons downstream the putative invertible promoters. We searched the protein sequences of these genes for known ARGs from the Comprehensive Antibiotic Resistance Database (CARD) (v3.0.7)^62^ using BLASTn (v2.10.0)^41^. The BLAST results were filtered using the parameters: -perc_identity 80, -evalue 1e-10, and -culling_limit 1. Resistance Gene Identifier (RGI, v5.0.0)^62^ was also used to predict known antibiotic-resistance elements using the following parameters: rgi main --t contig -a BLAST -n 8 -d wgs --local. ARGs located in the operon immediately downstream of the identified invertible promoters were kept for further analysis. Pairwise BLASTn searches were performed between each pair of contigs with putative IP-ARG from samples of the same individual. If multiple contigs from samples of the same individual likely originated from the same genomic region, only the longest contig was selected as a representative in further analysis.

### Identification of host species and mobile genetic elements

The identified contigs containing ARGs regulated by invertible promoters were searched with BLASTn against the NCBI nonredundant nucleotide (nt) database and the Unified Human Gastrointestinal Genome UHGG database^37^ with an e-value threshold <1e-10. If all best BLAST hits for a contig were from the same species, the species taxonomy was assigned to the contig. If there are ambiguous matches (hits from multiple species with the same top-match statistics), the lowest common ancestor of all the hits was assigned as the taxonomy of the contig. Next, we performed BLASTn to search for identical homologs (>99% identity) of the identified invertases and invertible regions against the 204,938 nonredundant genomes from the UHGG database. The identical homologs were grouped into corresponding classes of IP-ARG and the UHGG annotations for host species were examined to identify host species for IP-ARG.

To identify integrative and conjugative elements (ICEs) in different classes of IP-ARG, we searched the ICEberg^39,63^ and ImmeDB^38^ databases using BLASTn with an e-value <1e-10. We used ConjScan^64,65^ via a Galaxy web server (https://galaxy.pasteur.fr) to annotate genes involved in conjugation modules in ICEs.

### Genomic comparison and phylogenetic analysis

The bacteria genomes and genome comparisons were visualized with the R package genoPlotR. Genes were colored based on their predicted function. The function assignment of genes was first based on the prokka annotation. If a gene was annotated as “hypothetical protein” by prokka, we then performed BLAST search on the protein sequences of this gene against NCBI nr database as well as a small curated database of genes reported to be involved in polysaccharide biosynthesis^42^. The best hits were used to assign function to the genes.

To understand the evolutionary history of different classes of IP-ARG, a phylogenetic tree was constructed based on the invertases. The protein sequences of the invertase regulating ARGs regulated by invertible promoters identified in this study and those that have been previously reported^17^ were extracted. We also added invertases identified in the metagenomic assemblies based on PhaseFinder to the invertases dataset. If the invertase is located on a contig that lacks genomic context to determine if it is located on a mobile genetic element, the invertase was removed from further analysis. Redundant invertase genes were filtered using cd-hit with the 99% identity threshold^66^. Multiple alignments of protein sequences of the invertases were performed with MUSCLE (v3.8.31)^67^. The alignment results were analyzed in FastTree (v2.1.10)^68^ with default parameters to infer the phylogenetic trees. Only the invertase genes regulating functionally characterized genes or operons were included in the tree. *Bacteroidales* integrases^38^ were added to the alignments and used as the outgroup to root the tree. The phylogenetic tree was visualized using iTOL (https://itol.embl.de).

### Reporting summary

Further information on research design is available in the Nature Research Reporting Summary linked to this article.

## Supplementary information

Supplementary Information

Supplementary Data 1

Supplementary Data 2

Supplementary Data 3

Supplementary Data 4

Reporting Summary

## Data Availability

The human gut metagenomic data used in this study are downloaded from the NCBI. Accession numbers are listed in Supplementary Data 1. Metagenomic assemblies are available on the NCBI FTP site at https://ftp.ncbi.nlm.nih.gov/pub/mgx/IP-ARG_assemblies/. Nucleotide sequences of the identified contigs carrying ARGs regulated by invertible promoters can be accessed at https://ftp.ncbi.nlm.nih.gov/pub/mgx/IP-ARG_assemblies/IPARG_contigs.fasta.
