# Supplementary material for: Bacteroidales species in the human gut are a reservoir of antibiotic resistance genes regulated by invertible promoters
Source: NPJ Biofilms Microbiomes. 2022 Jan 10;8:1. doi: 10.1038/s41522-021-00260-1 (PMC8748976; doi:10.1038/s41522-021-00260-1)
Supplement: Supplementary file 1 — Supplementary Information [file 41522_2021_260_MOESM1_ESM.pdf]

Supplementary Information for

Bacteroidales species in the human gut are a reservoir of antibiotic resistance genes regulated by invertible promoters

Wei Yan, A. Brantley Hall, and Xiaofang Jiang*

* Corresponding author. Email: xiaofang.jiang@nih.gov

Supplementary Figure 1: Sequence alignment of IP-ARG genomic context in identified contigs from metagenomic assemblies with the known ICEs.

Supplementary Data 1: Human gut metagenomic samples analyzed in this study.

Supplementary Data 2: Information of the identified sequences containing ARGs regulated by invertible promoters.

Supplementary Data 3: The number of metagenomic reads supporting ON or OFF orientation of invertible promoters.

Supplementary Data 4: UHGG genomes carrying the homologs of the identified invertase genes mediating ARGs regulated by invertible promoters.

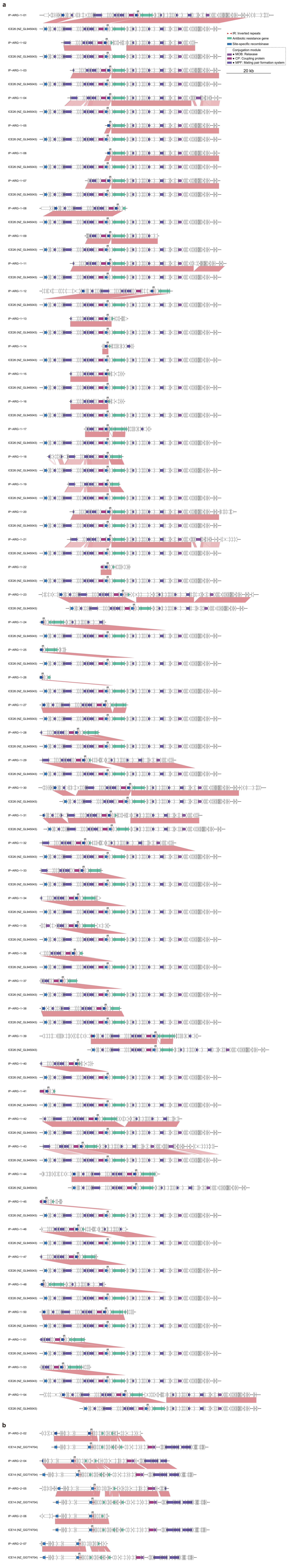

Supplementary Figure 1: Sequence alignment of IP-ARG genomic context in identified contigs from metagenomic assemblies with known ICEs. Only contigs that have not been shown in the Figure 2 are presented. Inverted repeats, site-specific recombinase, antibiotic resistance genes as well as conjugaion modules are colored. Orthologous genes are linked by pink connections between metagenomic contigs and known ICEs.
